# A Case of Robot-Assisted Ileocecal Resection for Ascending Colon Cancer with Ileocolic Vein Flowing into the Jejunal Vein: Case Report

**DOI:** 10.70352/scrj.cr.26-0029

**Published:** 2026-04-09

**Authors:** Kyoko Kobayashi, Masakatsu Paku, Hidekazu Takahashi, Takashi Honda, Naoki Sakamoto, Kei Hukumori, Juavijitjan Watsapol, Yuka Iwami, Satoshi Ishikawa, Kazuya Iwamoto, Shohei Takaichi, Tomofumi Ohashi, Yujiro Nakahara, Kohei Murakami, Tadafumi Asaoka, Takeshi Omori, Ichiro Takemasa

**Affiliations:** 1 Department of Gastrointestinal Surgery, Osaka International Medical & Science Center Osaka Keisatsu Hospital, Osaka, Osaka, Japan; 2 Department of Radiology, Osaka International Medical & Science Center Osaka Keisatsu Hospital, Osaka, Osaka, Japan

**Keywords:** ileocolic vein, robotic surgery, right-sided colon, ileocolic resection

## Abstract

**INTRODUCTION:**

Anatomical vascular variations are frequently observed in the right colon. The ileocolic vein (ICV) typically drains directly into the superior mesenteric vein (SMV). When vascular anomalies are present, surgical procedures often differ from standard techniques, which frequently increases the difficulty of the operation. Therefore, preoperative confirmation of vascular variations is essential. Here, we present a case of ascending colon cancer in which a rare variation in the ICV was diagnosed preoperatively.

**CASE PRESENTATION:**

An 84-year-old woman visited her physician complaining of hematochezia and was diagnosed with ascending colon cancer by colonoscopy. She was then referred to our hospital for surgical treatment. Preoperative CT revealed that the ICV had drained into the jejunal vein (JV) instead of the SMV. The patient underwent robot-assisted ileocecal resection. Intraoperatively, it was confirmed that the ICV drained into the JV, which ran on the dorsal side of the SMV, consistent with the preoperative imaging findings. The ICV was ligated at the level of the right SMV margin. No perioperative complications occurred, and the patient was discharged on POD 7.

**CONCLUSIONS:**

Recognition of this rare variation in ICV preoperatively enabled safe and successful robot-assisted ileocecal resection with D3 lymph node dissection. No reports of a patient with a vascular anomaly similar to this case who underwent surgery were published.

## Abbreviations

ARCV
: accessory right colic vein
CME
: mesocolic resection
GCT
: gastrocolic trunk
ICA
: ileocolic artery
ICV
: ileocolic vein
JV
: jejunal vein
MCA
: middle colic artery
MCV
: middle colic vein
SMA
: superior mesenteric artery
SMV
: superior mesenteric vein

## INTRODUCTION

The vascular anatomy of the right colon varies considerable variation.^1)^ Therefore, a precise understanding of the vascular anatomy is essential for right-sided colon cancer surgery. The ICV typically drains blood from the terminal ileum and the lower ascending colon directly into the SMV.^2)^ However, variations in the ICV are often observed, and recognition of this variation preoperatively is required to ensure safe surgical procedures.

Herein, we report a case of robot-assisted ileocecal resection that was safely performed by identifying a rare variation of the ICV associated with ascending colon cancer preoperatively.

## CASE PRESENTATION

**Fig. 1:**
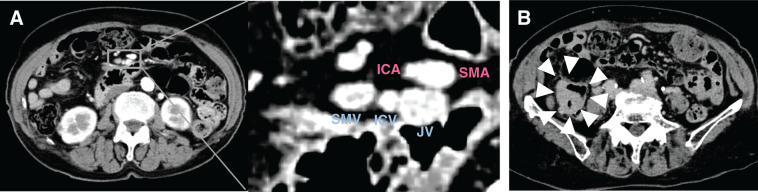
Contrast-enhanced abdominal CT findings. (**A**) CT shows ICV flows into the JV. (**B**) The arrowheads indicate the tumor in the cecum. ICA, ileocolic vein; ICV, ileocolic vein; JV, jejunal vein; SMA, superior mesenteric artery; SMV, superior mesenteric vein

**Fig. 2:**
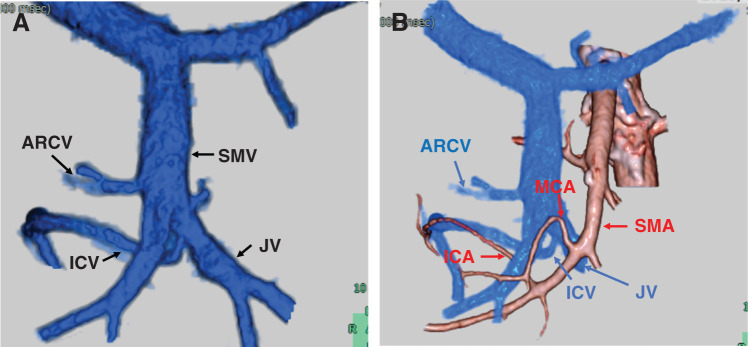
SYNAPSE VINCENT. (**A**) SYNAPSE VINCENT shows the branching of the ICV more clearly. (**B**) The ICV runs behind the SMA. ARCV, accessory right colic vein; ICA, ileocolic vein; ICV, ileocolic vein; JV, jejunal vein; MCA, middle colonic artery; SMA, superior mesenteric artery; SMV, superior mesenteric vein

An 84-year-old woman visited her local physician complaining of rectal bleeding. She was 133.8 cm tall and weighed 45.5 kg (BMI, 25.4 kg/m^2^). She had a history of diabetes, interstitial pneumonia, dyslipidemia, and hypertension. Lower gastrointestinal endoscopy revealed a type II tumor in the ascending colon. Based on the results of the biopsy, the tumor was diagnosed as a moderately differentiated tubular adenocarcinoma. Contrast-enhanced CT images of the chest and abdomen revealed wall thickening with contrast enhancement in the ascending colon, which was identified as the primary tumor. Enlarged lymph nodes were observed near the cancer site; however, no findings suggestive of distant metastasis were observed. To identify vascular anomalies, the CT images were analyzed in 3D using SYNAPSE VINCENT (V7.0.0003; FUJIFILM, Tokyo, Japan), confirming that the ICV runs cephalad to the ICA and dorsal to the SMV, and flows into the JV between the SMV and SMA (**Figs. 1** and **2**). Blood test results showed CEA and CA19-9 levels of 68.5 and 26, respectively. Based on these findings, the patient was clinically diagnosed with ascending colon cancer, stage IIIb (cT3N1aM0) according to the TNM classification, and underwent robot-assisted ileocecal resection and D3 lymph node dissection. The surgery lasted 172 min, with minimal blood loss. The final pathological diagnoses were pT4aN1bM0 and pStage IIIb.

We placed an 8-mm port in an inverted L-shape, 5-mm ports on both sides of the abdomen, and a 12-mm port at the umbilicus. The robot used was the Da Vinci 5 (Intuitive Surgical, Sunnyvale, CA, USA). Fenestrated bipolar forceps were placed in the first robotic arm, monopolar curved scissors in the third arm, and Cadiere forceps in the fourth arm. During lymph node dissection, the instrument in the third arm was exchanged for a Maryland bipolar forceps, and a double bipolar technique was employed.

**Fig. 3:**
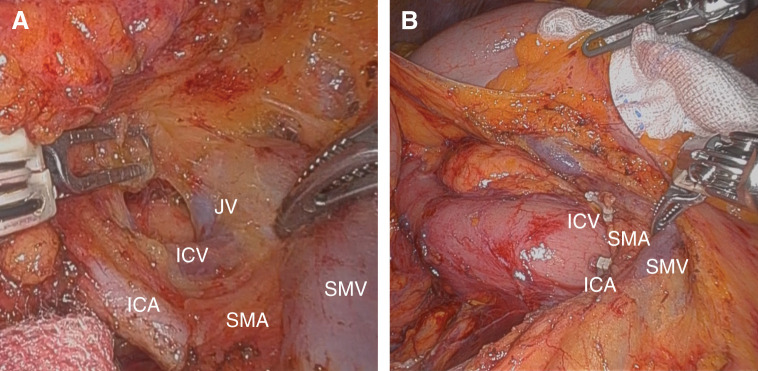
Intraoperative findings. (**A**) It shows that the SMA runs along the dorsal aspect of the SMV and gives off the ICA. The ICV runs along the dorsal aspect of the SMA. (**B**) CME and complete lymph node dissection was performed. CME, mesocolic resection; ICA, ileocolic vein; ICV, ileocolic vein; JV, jejunal vein; SMA, superior mesenteric artery; SMV, superior mesenteric vein

**Fig. 4:**
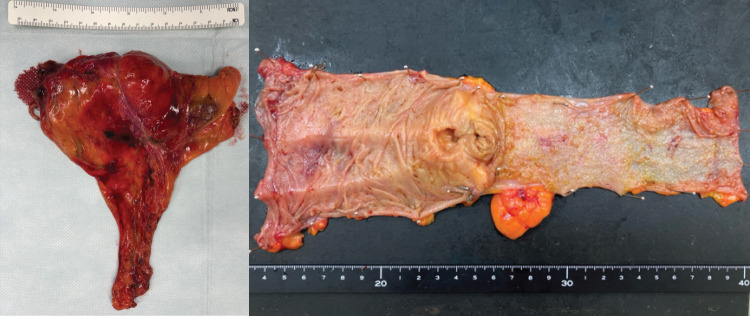
The resected right hemicolon specimen.

The surgery involved a retroperitoneal approach, with the right colonic mesentery dissected from the retroperitoneum, and the duodenum and pancreatic head dissected from the right colonic mesentery. The approach shifted to the medial side, with the tail margin of the dissection determined using the ICA pedicle as a guide, exposing the anterior surface of the SMV. The left margin of the SMV was designated as the left dissection range. The ICA, which runs along the dorsal side of the SMV, was examined. As shown in preoperative vascular imaging, the ICV runs along the dorsal side of the SMV cephalad to the ICA, joins the JV on the left side of the SMV to form a common trunk, and flows into the left side of the SMV. Therefore, the ICA was first transected at the level of the right margin of the SMV and the ICV was transected at the same level (**Fig. 3**). The MCA and the ARCV was confirmed, the surgical trunk was cleared, and CME was performed (**Fig. 4**). The MCV was not detected on preoperative CT or during surgery. The right colon was removed through the umbilical wound and a functional end-to-end anastomosis extra-corporeally was performed. The patient had no postoperative complications and was discharged 7 days postoperatively.

## DISCUSSION

In right hemicolectomy for right colon cancer, CME is essential and requires careful dissection along the mesocolic plane and meticulous vascular management on the central side.^3)^ Significant results from the Erlangen team showed a reduction in the 5-year local recurrence rates from 6.5% down to 3.6%.^4)^ However, performing CME for right hemicolectomy is challenging due to vascular anomalies. This can lead to bleeding during surgery or misidentification of blood vessels; therefore, identifying vascular anomalies preoperatively is essential, particularly in cancer surgeries involving lymph node dissection. In particular, in right-sided colon cancer surgery, not only vascular anomalies but also complex relationships with surrounding organs, such as the pancreas and duodenum, require careful attention to prevent bleeding due to pancreatic or vascular injury. These make surgeries difficult and raise the risk of complications. A retrospective analysis of Japan's National Clinical Database showed that the 30-day postoperative mortality rate for right hemicolectomy was 1.2%. Blood transfusions were performed in 2.8% of cases, which is by no means a low frequency.^5)^ Therefore, preoperative vascular imaging has been reported to be helpful in right-sided colon cancer surgery.^2)^ In this case, as shown in **Fig. 2**, preoperative vascular imaging using 3D image analysis allowed us to preoperatively identify the spatial relationship between the ICA and the ICV, the course of the ICV, and the manner of ICV confluence.

The mobilization and resection is in the right colon, the ICV is present in 100% of the cases and typically flows directly into the SMV, making it an essential landmark during surgery for right colon cancer.^1)^ By contrast, the ICV is relatively prone to variations such as standard trunk formation with the right colonic vein or middle colonic vein, inflow into the GCT, or abnormal confluence with the SMV, with a reported frequency of 10%–30%.^6,7)^ In this case, the ICV passed posterior to the SMV and flowed into the JV between the SMA and SMV, while its common trunk flowed into the SMV from the left side. This vascular variation was very rare; to our knowledge, this venous branching pattern has previously been described only in a cadaveric study by Kuzu et al.,^8)^ and no reports have documented its occurrence in a clinical setting. Furthermore, we found no published cases describing the surgical management of this specific anomaly, whether open or laparoscopic. In this case, the significance lies not only in the use of robot-assisted surgery but also in the preoperative evaluation of the vascular anatomy using the SYNAPSE VINCENT imaging system, which enabled safe surgical management.

Robot-assisted surgery offers several advantages in cases where the ICV drains into the JV rather than the SMV, including enhanced instrument dexterity, a stable 3D magnified view, and precise motion control. Lymph node dissection was performed using the double bipolar technique with a Maryland bipolar forceps in the right hand. This method minimizes thermal damage to the surrounding organs and blood loss compared with monopolar scissors.^9,10)^ These features enable safe dissection and identification of complex and fragile venous anomalies.

Especially in patients with vascular anomalies, it is crucial to avoid venous injury by understanding vascular course preoperatively using 3D-CT, early identification of the SMV and the JV, and avoiding blind dissection based solely on standard anatomical assumptions.

## CONCLUSIONS

In conclusion, vascular reconstruction enabled preoperative recognition of vascular anomalies, allowing the safe implementation of robot-assisted ileocecal resection and D3 lymph node dissection in patients with ascending colon cancer accompanied by a rare vascular anatomical abnormality in which the ICV flows into the JV. Understanding vascular anomalies preoperatively is essential for selecting a safe and appropriate approach that could perform safe and radical CME for right-sided colon cancer.
